# On the Diagnosis of Syphilis

**Published:** 1806-01

**Authors:** 


### *To the Editors of the Medical and Physical Journal.*

GENTLEMEN,

THE following interesting Case is not presented to the public as a specimen of any thing novel, but to call the attention of medical men to a very important part of the profession, viz. the diseases resembling syphilis. Upon this subject some valuable information has been lately given to the world, and happy would it be for mankind, if in the general treatment of this disease, a due and cautious investigation of its progress, character, and appearances were first made, before its treatment was determined upon; we should not then be witness to the sad number of mutilated victims, who either have fallen a sacrifice to to the daring attempts of ignorant empiricism, or to the rash administration of the mineral specific; and it may well be doubted, whether more sacrifices are not annually made to the injudicious administration of mercury, than to all the combined effects of the morbiferous poisons. The subject of the present paper was a gentleman, for whom I was consulted in the early part of the present year, on account of an excoriation on the side of the prepuce; the history of the case was this: His mouth had been for some time in an indifferent state, probably owing to the very impure quality of the atmosphere, which, from the nature of his situation, he was then confined to.

In the early part of January he had a connection; from this, however, no visible morbid symptom arose till five or six weeks after, when a slight irritation was felt upon the prepuce; this was soon followed by a sore, extending from the middle of the left side of the prepuce to the frænum, and succeeded by an enlargement of the glands in each groin. Upon viewing accurately the sore, whose surface exhibited little of the characteristic marks of the true venereal chancre, and particularly attending to the progress of the case, and the length of time that had elapsed between the connection and the appearance of the sore, I pronounced it not to be venereal, and directed the most simple applications to be made, as, the saturnine solution, or a poultice of milk and bread; this plan was adhered to for some time, when it not appearing to form a disposition to heal, and considering that it might probably be owing to the present indifferent state of his health, recommended a mild mercurial wash, which in a short time completely healed it.

Nothing now whatever appeared, till early in May, when I was again consulted by the same gentleman, on account of an uneasiness in his throat; upon examination I distinctly perceived a slight ulceration upon the tonsils, accompanied with a degree of erysipelatous redness. The ulcerations kept gradually, but superficially, spreading for about a week, when an eruption of purple spots took place upon the skin, and at the same time the glands in the groin continued greatly enlarged.

Confident, however, from the appearance of the sore, and particularly from the history of its progress, that it was not venereal, I recommended the use of mild deter-gent gargles, and a plentiful quantity of Red Port daily. From this time, however, a uniform increase of the symptoms took place, the eruptions became more vivid,

 and and the tonsils, although, extensively ulcerated and sloughy, did not appear to penetrate deep, or to assume the thickened ragged edge. The case at this time becoming of some importance, I was desirous of my patient having further advice. It was accordingly agreed to consult Mr. Pearson, who upon hearing the history of the case, and minutely examining the appearances, gave it as his opinion, that it was not venereal; in addition to the plan before recommended, he advised a plentiful use of the Peruvian bark with elixir of vitriol, and the application of the oxymel eruginis to the tonsils. From this period to the beginning of July, a slow and somewhat interrupted progress was made; the ulcerations had extended from the tonsils up the inferior edge of the velum palatinum, when at this period the tonsils assumed a disposition to heal, but again quickly ulcerated. Little benefit having resulted from the means hitherto made use of, and his health beginning to suffer, it was determined that he should discontinue all medicine and repair into the country. The symptoms which had hitherto been characterized by a very slow progress, now assumed a much more rapid increase; the ulcerations spread on one side of the velum palatinum, crossed the uvula, and completely affected the other; a deep ulceration took place upon the tongue, and the copper coloured eruptions became larger, and more universally diffused.

The conviction, however, of the peculiar character of the primary symptoms, still supported me in the belief, that, although a most obstinate poison was secreted and thrown into the system, yet, that it was essentially different in its true specific properties from the venereal poison; in this I was shortly corroborated, for a fortnight had not elapsed, before a sudden and almost an entire disappearance of the eruption took place: this is a character which most decidedly marks the spurious syphilis from the genuine, as will be shortly observed at the conclusion of the case. In a few days the eruptions again appeared, and continued to the end of three weeks, at which time the progress of the poison appeared to have arrived at its achme; the ulcerations in the throat began to subside, their sloughy surface to look more healthy and granulate, and the eruptions gradually disappeared. Since that period some slight fluctuations have taken place, sometimes all the symptoms being nearly exhausted and then appearing to resume a slight morbid action; this was particularly the case in the early part of September, when a supera superficial ulceration re-attacked the right tonsil, and affected the left with small scattered specks. At this period I recommended a strong decoction of bark, combined with a small quantity of sarsaparilla and burnt sponge, and in each dose of the above, from ten to sixteen drops of the muriated barytes; under this medicine every symptom has subsided, and the patient's health nearly re-established.

Thus has a most interesting case, which lasted with very ambiguous symptoms from the beginning of May to the early part of October, finally terminated without *one single particle of mercury*, and in a constitution where, probably, a complete course of that mineral would have proved fatal.

### Remarks.

It has been justly lamented, by accurate and eminent practitioners, that no criterion could be formed, at least by appearance, of the difference between the true and spurious venereal sore; this, I confess, in numerous cases, is a difficult and almost impossible thing, and whoever solely trusts to the appearance of either a primary or secondary affection, without being guided by the history of the case, will be subject to perpetual error. The above case is a striking fact, elucidating this proposition, for I am convinced, that let the sagacity of that practitioner have been what it would, his judgment must have been most palpably deceived had he depended upon the appearance alone. The venereal poison, as far as the experience of many centuries confirm, is a virus, which, when once received into the constitution, no power that it posseses is capable of expelling; and, to quote the words of Mr. Pearson, (whose great judgment and superior abilities, particularly in this part of his profession, will confer a lasting honour upon his character), "That when once the venereal poison is received into the system, it will go progressively forward to the destruction of the animal, unless checked by the use of mercury." This therefore forms the grand essential difference between the two poisons, that the former will ultimately destroy, except checked by medicine; the latter fluctuates, and will at last exhaust itself. But, besides this distinction of its principle, a minute investigator, who has an opportunity of seeing the primary affection, will still further discriminate from the *character*, and more particularly from the account of the *progress* of the case. This was one of the circumstances that that appeared to decide in the present case; for, First, to imagine that a true venereal sore could take place several weeks after exposure, and particularly when a due attention had been paid to cleanliness, was a fact which militated against all that we ever knew of the venereal disease. Secondly, the nature and appearance of the sore did not correspond with the venereal character, its base was not sloughy or deep, it did not assume the thickened edge, and the slight surrounding induration that was left gradually subsided. Thirdly, the glands in the groin, which had arrived at a considerable enlargement, as soon as the sore was healed ceased to increase, and although it is not absolutely necessary that every true venereal enlarged gland should suppurate, yet the probability that they are spurious is in their favour, particularly when they gradually diminish without the use of mercury; and, lastly, the slight depth which the secondary ulcerations made, the regular edge, and the fluctuating appearance of the spots, were criteria, which the most sceptical observer cannot controvert.* I am, &c.

*November* 30, 1805. W. S. C.

* Medical practitioners in general, on this side the Tweed, appear to be ignorant of the disease so well known in Scotland by the name of Sibbens or Sivvens, of which the above is, probably, an instance. The interesting question, whether this is to be considered as an hybrid disease, or as a new species not yet distinguished by Nosologists, must be left to future observation. All communications tending to elucidate this important subject must be considered as most interesting, not only to medical men, but the world at large. Ed.

